# Understanding Democracy and Development Traps Using a Data-Driven Approach

**DOI:** 10.1089/big.2014.0066

**Published:** 2015-03-01

**Authors:** Shyam Ranganathan, Stamatios C. Nicolis, Viktoria Spaiser, David J.T. Sumpter

**Affiliations:** ^1^ Department of Mathematics, Uppsala University, Uppsala, Sweden.

**Keywords:** big data analytics, mathematics, predictive analytics

## Abstract

Methods from machine learning and data science are becoming increasingly important in the social sciences, providing powerful new ways of identifying statistical relationships in large data sets. However, these relationships do not necessarily offer an understanding of the processes underlying the data. To address this problem, we have developed a method for fitting nonlinear dynamical systems models to data related to social change. Here, we use this method to investigate how countries become trapped at low levels of socioeconomic development. We identify two types of traps. The first is a democracy trap, where countries with low levels of economic growth and/or citizen education fail to develop democracy. The second trap is in terms of cultural values, where countries with low levels of democracy and/or life expectancy fail to develop emancipative values. We show that many key developing countries, including India and Egypt, lie near the border of these development traps, and we investigate the time taken for these nations to transition toward higher democracy and socioeconomic well-being.

## Introduction

As more data becomes available, we increase our understanding of processes and dynamics we observe in societies. One phenomenon that has received some attention in the economics and political science research is development traps. Under certain conditions countries can get stuck in these traps and experience a period of stagnation of the socioeconomic development on a low level. Data provided by the World Bank or the United Nations (UN) may give some insight into this phenomena. Data mining and machine learning techniques,^1,2^ for instance, could facilitate predictions about the risk of countries getting trapped in low development, and the time required for countries to move out of development traps. This can be a useful approach if predictions are the ultimate goal. However, such an approach is limited in terms of providing understanding^2–6^ of the dynamics and mechanisms of development traps.

Data scientists are now discussing ways to extend or find new methods in machine learning that would allow modeling of causality, detecting mechanisms or including social and economic theory in data science approaches.^7–9^ In an earlier article,^10^ we propose a data-driven dynamical systems approach to deriving differential equation models from panel data. Our approach is inspired by machine learning approaches, to the extent that model building is data driven. However, our approach differs from a pure machine learning approach, in that we do not merely fit models to make best possible predictions but derive a set of equations that describe the underlying processes. The hope is that these equations will aid understanding of the underlying process.

**FIG. 1.**
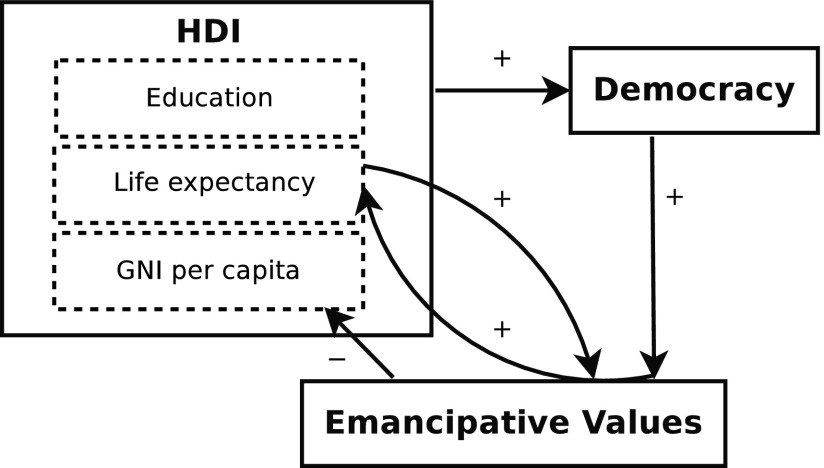
Dynamic model of interactions between socioeconomic indicators, democracy, and cultural values.

One question where understanding is just as important as prediction is in the change of cultural values, development, and democracy.^11–14^ In Spaiser et al.^15^ we applied our method to look at how democracy, socioeconomic development, and cultural values have changed across different countries in the last 30 years. We fitted differential equations for the rate of change of six indicators as a function of the level of the indicator itself and the level(s) of other predictors in the previous year. The result of this work was a general dynamical model of the interactions between democracy, cultural values, and socioeconomic indicators that provided the best fit to the available data. Figure 1 summarizes the dynamic relations we found between the various indicators.^15^ In brief, we found that a critical level of the Human Development Index (HDI) triggers democratization and then the emancipation of the population. However, while emancipative values contribute to equal access to education and a healthy life, they do not lead to further accumulation of wealth. Thus once countries reach high levels of democracy and emancipation, they tend toward equilibrium in terms of economic growth.

The next step, and the question we address here, is to use our model to understand the underlying process of human development. This is an essential step if we are to address the issue of mechanisms and causality.^7–9^ It is also essential if we are to disentangle the consequences of the interactions between values, democracy, and development shown in Figure 1. While this flow diagram indicates positive and negative feedbacks, the picture becomes complicated when we consider the underlying nonlinear differential equations (presented in detail below). From these we can see various threshold effects, where interactions change sign depending on the level of interacting variables. In particular, these thresholds suggest the possibility of “development traps,” where countries can become “trapped” with low levels of democracy and/or socioeconomic development. The question is how these traps interact to determine the course of socioeconomic and political development in the world.

Development traps are widely discussed in the economics literature. For instance, Nelson^16^ and Azariadis and Drazen^17^ examined causes such as endogenous population growth or technological externalities with a “threshold” property, for persistently low rates of growth or relatively low levels of economic development of countries. More recently, Sachs^18^ analyzed causes for poverty traps in terms of misbalances in different types of capital, for instance, undersupply of human capital in terms of education.^19^ A poverty trap is generally seen as a self-reinforcing mechanism that causes poverty to persist, usually from generation to generation.^20^ Low growth and poverty traps are closely linked, since they represent different perspectives on underdevelopment.^16,21^

Rather than concentrating solely on economic growth traps, we look at development traps with respect to both democratization and socioeconomic development. Our starting point is the empirical relationships summarized in Figure 1, and our aim is to clarify the implications of these relationships in terms of development traps. To do this we employ two tools. The first is dynamical systems style^22^ mathematical analysis of the nonlinear equations that best fit the data. The second is stochastic simulation of the equations. We model inherent variability in development using independent Gaussian noise variables and account for the unpredictability by performing stochastic integrations to obtain estimates of future probability distributions in the socioeconomic, political, and cultural values indicators.

## Data and Methods

We used six different indicators in our analysis: four socioeconomic indicators and one democracy and one cultural values indicator. The analysis has been done for the time period 1981–2006 for 65 countries (for details on data availability, see Table 1 and Spaiser et al.^15^). HDI, *H*, is our main socioeconomic indicator. HDI is a composite index, consisting of measures for education (composite measure of average years of schooling and expected years of schooling) *I*, life expectancy (in years) *L*, and gross national income (GNI) per capita (in $) *G*^23,24^ As in our earlier article, we perform analysis with HDI and additionally with the single HDI components.

**Table 1.** **Six Development Indicators We Use in Our Analysis and Other Details About the Data**

*Indicator*	*Range*	*Components*	*Source*	*Years*	*Countries*
Human-rights democracy	0–1	Political rights score, civil liberties score, human-rights performance scores	Freedom House, Cingranelli & Richards Human Rights Data Project (CIRI)	1980–2006	187
Emancipative values	0–1	—	World Value Survey (www.wvsevsbd.com)	1981–2011	65
Human Development Index	0–1	UN education index, life expectancy, GNI per capita	UNDP (http://data.un.org)	1980–2012	193
log GNI per capita	5–12	—	World Bank (http://data.worldbank.org)	1980–2012	213
United Nation education index	0–1	Means years of schooling, expected years of schooling	UNDP (http://data.un.org)	1980–2012	193
Life expectancy	42–83	—	World Bank (http://data.worldbank.org)	1960–2012	213

GNI, gross national income.

In our article, *D* represents human-rights democracy; that is, the democracy index consists of indicators measuring civil liberties and political rights provided by Freedom House^25,26^ and of human right indices provided by the Human Rights Data Project.^27^ Finally, cultural values refer to the World Values Survey^28^ emancipative values index *E*, which measures preferences for “decision making freedom of the individual human being and the equality of all human beings in this decision-making freedom”^29^ on the aggregate level.

These six indicators were chosen because the analyses in this article build on results we have published in an earlier article.^15^ Originally, the selection of these indicators was inspired by the Human Development Sequence Theory^11,12^ that assumes that socioeconomic development, cultural change, and democracy are interrelated. We have tested alternative indicators for democracy (effective democracy), cultural change (self-expressive values), and socioeconomic development (GDP per capita, female education) in our two earlier article, and the six indicators that were selected for analyses in this article proved to be best predictors. The results are also consistent over different indicators, with some exceptions for the democracy indicators, which we have discussed in the previous article.^15^

The variables *H*, *D*, *I*, and *E* were scaled from the original data to be values between 0 and 1. The other two variables *G* and *L* were rescaled to values between 0 and 1. *L* is restricted to be positive and scaled such that a value of 1 corresponds to actual life expectancy of 100 years. Realistic values of *L* are between 0.3 and 0.9. For *G* we first computed the logarithm of a country's per capita GNI, which results in a *G* range between 5 and 12. In the second step we rescale *G* by dividing the logarithmic values in the data by the maximum 12. As a result *G* ranges between 0.46 and 1, which corresponds to around $260 and $160,000, respectively (for more details on scaling and data generally, see Spaiser et al.^15^).

In our previous article, we already identified relationships between the five variables. Specifically, we fit the changes (*dG*, *dl*, *dL*, *dD*, and *dE*) as differential equations with polynomial terms of the indicator variables themselves, that is, *G*, *I*, *L*, *D*, and *E*. For the five variables, these were

\begin{align*} \frac { dG }  { dt } = 0.002 \frac { G ( t ) }  { E ( t ) } \tag { 1 } \end{align*}

\begin{align*} \frac { dl }  { dt } = 0.007 \tag { 2 } \end{align*}

\begin{align*} \frac { dL }  { dt } = 0.028E ( t ) ( 1 -
\frac { 0.887 }  { L ( t ) } ) + \frac { 0.004 }  { L ( t ) } \tag
{ 3 } \end{align*}

\begin{align*} \frac { dD }  { dt } = 0.11 D ( t ) ( G ( t ) I ( t ) - 1.08D ( t ) ) + 0.025G ( t ) ^2 \tag { 4 } \end{align*}

\begin{align*} \frac { dE }  { dt } = 0.028D ( t ) ( L ( t ) - 0.585 ) \tag { 5 } \end{align*}

The methodology used in the model selection is described in detail in Ranganathan et al.^10^ and the steps taken to obtain these specific models are described in Spaiser et al.^15^

In this article, we first use phase portraits based on the equations above to identify different patterns of behavior such as trap regions where the yearly changes slow down to very small values. Second, we look at stochastic integrations of the model.

In order to incorporate stochastic dynamics, we use discrete time versions of equations and include a noise term estimated from the data. For a simplified two-variable model, these are Equations (6) and (7) below, while for the five-variable model these are Equations (11)–(15) below. The deterministic terms in these models are precisely the same as described above, although now expressed in discrete time. The noise term in each variable at each time step is a constant multiplied by an independent normally distributed random variable with mean zero and standard deviation 1. The noise constant is the standard deviation of the error in the model fit, that is, the square root of the residual sum of squares divided by number of observations.

The stochastic integrations were performed starting from a set of four different initial conditions in the five variables corresponding to values for Egypt, India, Jordan, and Ukraine in 2006. At each time step we predict the yearly change using the model and add this to the current levels in the indicator variables along with independently generated observations from Gaussian noise variables corresponding to the description above. Since *H*, *D*, *I*, and *E* only take values from 0 to 1, we force the boundary conditions so that any value over 1 is taken to be 1 and any value below 0 is taken to be 0. The lower boundary condition also applies to the variable *L* and we assume that it applies also to *G*, effectively imposing a minimum GNI per capita of 1 dollar.

## Results

### Democracy and development

Before we deal with the full model from Figure 1, we start by analyzing the interactions of just two indicator variables: human-rights democracy and HDI. This analysis will pave the way for the more complex model established between the five indicator variables in the full model. The best fit model relating democracy and HDI is

\begin{align*}D_{t + 1} - D_t = 0.0709 \ H_t^2 - 0.0658 \ D_t + 0.08 \ \epsilon_D \tag{6}\end{align*}

\begin{align*}H_{t + 1} - H_t = 0.0045 + 0.004 \ \epsilon_H \tag{7}\end{align*}

The noise terms *ɛ_D_* (*t*) and *ɛ_H_* (*t*) are independent normally distributed random variables with mean 0 and standard deviation 1 and scaled by the estimated error standard deviation. All parameters are estimated from the data as described in the Data and Methods section.

The model of HDI is straightforward to interpret. HDI grows at a constant rate, plus or minus a random term, and is unaffected by democracy. The democracy equation is more complicated. Democracy only increases in countries where

\begin{align*}H > 0.96 \sqrt{D} \tag{8}\end{align*}

For smaller values of *H*, *D* decreases and we say that a country with these values is caught in a democracy trap. Until socioeconomic development reaches a sufficient level, democracy does not tend to develop in countries below this threshold.

The democracy trap condition [Eq. (6)] is quantitative, since it is estimated directly from the available data. It does not imply that every country below the line fails to increase in terms of democracy. Rather, it reflects the fact that over the last 30 years those countries that have not fulfilled the HDI condition have generally experienced decreases in democracy, while those above it have experienced increases. We can, and later in this article do, speculate why such a trap exists, but for now we simply note that it is an empirical pattern in the available data that can be summarized in terms of a single equation.

For this two-variable model, we can go on to obtain a full solution to the equations, in the absence of noise. Equation (7) can be solved directly to give

\begin{align*}H_t = 0.0045t + H_0 \tag{9}\end{align*}

where *H*_0_ is the initial level of HDI. Replacing Equation (9) into Equation (6) and solving the continuous time version of the equation we get (10).

\begin{align*}\begin{split}D_t =& 1.08 \ H_0^2 - 0.15 H_0 + 0.01 ( 1 + H_0t ) \
\\\quad -& \ 6.63 \times 10^{ - 4} t + 2.18 \times 10^{ - 5}\,t^2 \
\\\quad +& \exp (  - 0.066t ) \ ( D_0 - 1.08 \ H_0^2 + 0.15 H_0 -
0.01 )\end{split} \tag{10}\end{align*}

where *D*_0_ is the initial level of democracy. Equation (10) allows us to determine the time needed for the system to reach a particular value of *D* given a particular initial condition, *H*_0_ and *D*_0_.

**FIG. 2.**
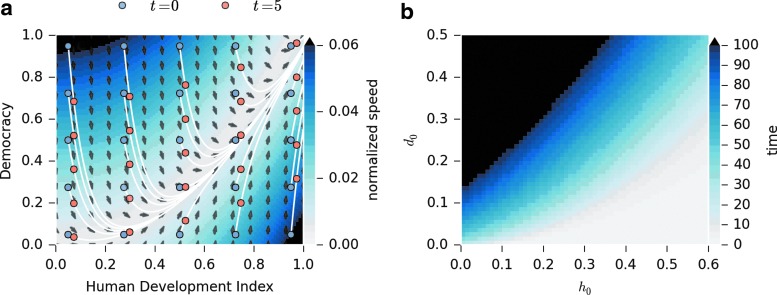
**(a)** Phase plane for the explicit solution of the two-variable model. The arrows represent the direction of the yearly change in the two variables, while the magnitude of change is shown by the color in the heatmap. Some sample trajectories are also shown with points 5 years apart depicted by blue and orange circles to show the expected change in the variables over a 5-year period starting from different initial conditions. **(b)** Heatmap showing time required to increase the democracy index by 20% as a function of initial conditions *H*_0_ and *D*_0_ [numerical solution of *t* vs. *Dt* of (10)].

Figure 2a provides a full-phase portrait of Equations (6) and (7) (in the absence of noise). There are two time scales involved in the evolution of *H*, and *D_t_*. First the trajectories evolve rapidly toward a slow manifold, defined by the democracy trap condition $H = 0.96 \sqrt{D}$. Then on the slower time scale, democracy and HDI evolve upward along this manifold. Figure 2b gives the time needed for a country to increase its democracy index by 20% as a function of initial conditions *H*_0_ and *D*_0_.

To provide a specific example, we take initial conditions corresponding to India in 1990, the first year the data is available (*H*_0_=0.404, *D*_0_=0.281 in 1990). We can ask how many years it will take for this country to double its democracy index and reach *D*=0.6. Solving numerically for *t*, we obtain *t*≈90 years. The model thus predicts that given its democracy level in 1990, India is expected to reach a level of 0.6 in 2080, which is close to that of Argentina in 2006. At the same time, the HDI will also double and reach *H*=0.81.

**FIG. 3.**
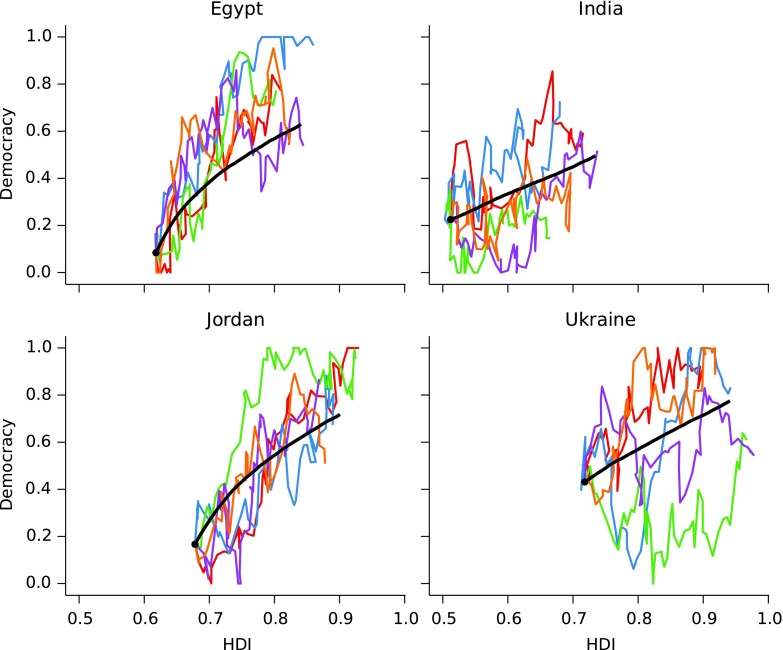
Stochastic integrations over a period of 50 years for initial conditions corresponding to Egypt, India, Jordan, and Ukraine. Average over 10,000 realizations (in black) and 5 different unique realizations (in color). Noise variances at each time step (corresponding to each year) are based on modeling error as specified in Equations (6) and (7).

The prediction that Indian democracy will grow slowly does not take in to account intrinsic and external noise or variability in the process of democratization and socioeconomic development. Noise and uncertainty is of course inherent in this process. While it is impossible to account completely for the uncertainty in future events, we can use the error in our model fit to account for some of this variability. Figure 3 shows example evolutions of a stochastic integration of Equations (6) and (7), including the noise term, for four different initial conditions corresponding respectively to Egypt, India, Jordan, and Ukraine.

**FIG. 4.**
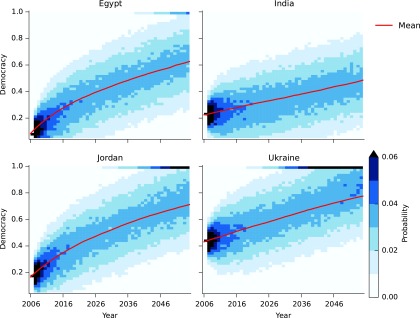
Heatmaps showing the evolution of the distribution of *D* values in a set of 10,000 stochastic simulations of the model for Egypt, India, Jordan, and Ukraine starting from 2006. Noise variance at each time step (corresponding to each year) based on modeling error.

Figure 4 shows a range of different possible democracy outcomes for India over a 50-year time scale, from long periods with continued low levels of democracy to increases to levels comparable with United States in 2006. For Ukraine, and also Egypt and Jordan, democracy levels comparable with countries in Western Europe, that is, greater than about 0.8, are plausible outcomes within 50 years. These are seen as the darker band at the top of Figure 4. In general, the noise in democracy is large, making it difficult to make reliable predictions using the two-variable model.

### Including HDI components and cultural values

The full model includes five variables. HDI is replaced by its three compound variables: GNI per capita *G_t_*, education *I_t_*, and life expectancy *L_t_*. We also add emancipative values, *E_t_*, as measured by the World Values Survey. The best-fit five-variable model is given by

\begin{align*}G_ { t + 1 } - G_t = 0.002 \frac { G_t }  { E_t } + 0.0042 \varepsilon_G ( t ) \tag { 11 } \end{align*}

\begin{align*}I_{t + 1} - I_t = 0.007 + 0.0053 \varepsilon_I ( t ) \tag{12}\end{align*}

\begin{align*}L_ { t + 1 } - L_t = 0.028E_t ( 1 - \frac {
0.887 }  { L_t } ) + \frac { 0.004 }  { L_t } + 0.0033
\varepsilon_L ( t ) \tag { 13 } \end{align*}

\begin{align*}D_{t + 1} - D_t = 0.11 D_t ( G_t I_t - 1.08 D_t ) + 0.025 G_t^2 + 0.083 \varepsilon_D ( t ) \tag{14}\end{align*}

\begin{align*}E_{t + 1} - E_t = 0.028 D_t ( L_t - 0.585 ) + 0.0062 \varepsilon_E ( t ) \tag{15}\end{align*}

where *ɛ_G_* (*t*), etc., are Gaussian random variables with 0 mean and variance 1. All parameters including the noise constants are estimated from the data as described in the Data and Methods section.

There are several insights that can be gained directly from these equations. First, the values for all the components of HDI generally increase over time or reach a stable equilibrium. In the case of life expectancy, this equilibrium is around 90 years old. Two of the HDI components, GNI and life expectancy, interact with emancipative values. This interaction is different for GNI, where countries with high emancipative values experience slow economic growth, than for life expectancy, which increases with emancipative values.

**FIG. 5.**
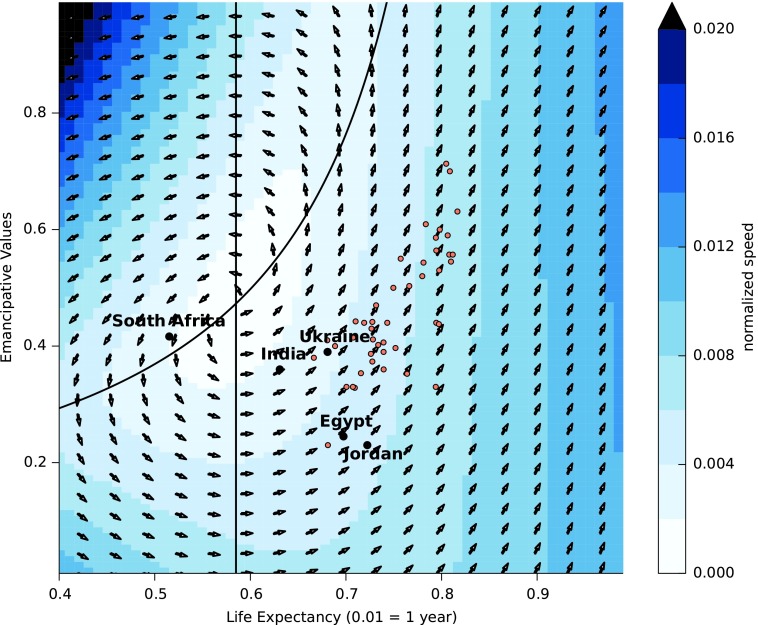
The phase portrait of *L* and *E*. The arrows show the direction of yearly change in the (*L,E*) vector and the color shows the magnitude of change (assuming *Dt*=1). The black curve corresponds to the solutions of *Lt+1*=*Lt* (assuming noise is zero), while the straight vertical line corresponds to *Et+1*=*Et*. The steady state at (*L,E*)=(0.585, 0.473) is a saddle node. The scatterplot points show data for all countries in the dataset in the year 2006. The four sample countries, Egypt, India, Jordan, and Ukraine, are highlighted. Additionally, South Africa, which appears to be an outlier, is highlighted.

Democracy and emancipative values both have potential development traps. In the case of emancipative values, *E* increases only if *L*≥0.585; that is, life expectancy is greater than 58.5 years. Figure 5 shows how *L* and *E* interact. The point (L*, E*)=(0.585, 0.473) is a steady state for this pair of equations in the absence of noise. Near to the solution of *L_t_*_+1_=*L_t_*, that is,

\begin{align*}E = \frac { 1 }  { 7 ( 0.887 - L ) } \tag { 16 } \end{align*}

the change in both *E_t_* and *L_t_* is small. It is here we can think of emancipative values being caught in a trap, although a temporary one. The scatterplot of data shows that in recent history most countries are moving away from the right edge of the “trap” region. The exceptions to this rule tend to have decreasing emancipative values. India and Ukraine are both in a region where change is slow. Eventually, countries will move away from this region, but this development happens slowly (velocity of change is color in Fig. 5). In the case of South Africa, we would predict a decrease in emancipated values before an increase is seen again.

To calculate the time it takes countries to increase their indicator values by a certain amount, we can do a further analysis of the system of equations. The steady state (L_*_, E_*_)=(0.585, 0.473) is a saddle node, since one eigenvalue of the Jacobian is positive and given by 0.028*D* and the other eigenvalue is given by −0.0145. Countries to the right of the characteristic curves (which show *L_t_*_+1_=*L_t_* and *E_t_*_+1_=*E_t_*) in Figure 5 continuously increase in both *L* and *E*.

**FIG. 6.**
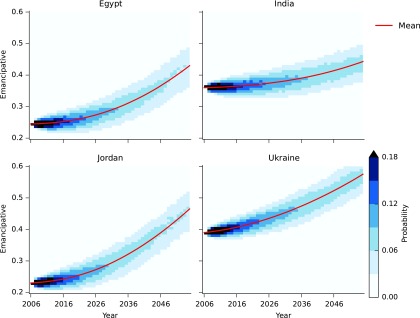
Heatmaps showing the evolution of the distribution of *E* values in a set of 10,000 stochastic simulations of the model for Egypt, India, Jordan, and Ukraine. The starting time of the simulation for each country is 2006.

Using the eigenvalues we can infer the time needed to increase the values of *E* and *D*. The positive eigenvalue 0.028*D* is the rate at which countries near to the steady state (L*, E*)=(0.585, 0.473) move away from it. Thus, countries with high values of democracy *D* move faster out of the trap region than countries with small *D*. Stochastic simulations of the model for Egypt, India, Jordan, and Ukraine show how these countries accelerate out of the trap (Fig. 6). Increases in emancipative values are slow over the first 20 years, especially in India and Ukraine, which are close to the trap region, but then accelerate. The variation between simulations is relatively small and these predictions are stable in the presence of noise.

The dynamic for democracy is similar to the two-variable model in the previous section, but now with an interaction between GNI and education. Specifically, *D* increases when

\begin{align*}D < G ( I + \sqrt{I^2 + 0.98} ) / 2.16 \tag{17}\end{align*}

**FIG. 7.**
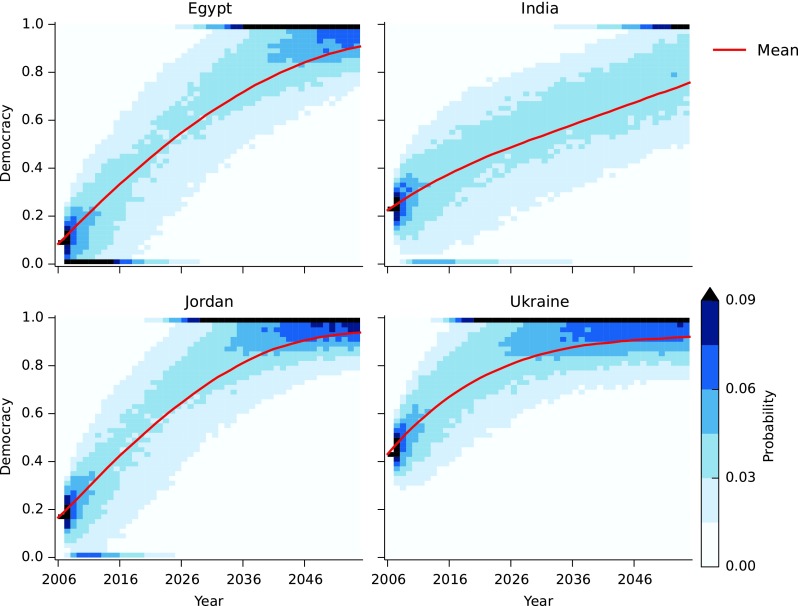
Heatmaps showing the time evolution of the distribution of the values of democracy *Dt* for Egypt, India, Jordan, and Ukraine starting from 2006.

Democracy requires both high levels of education and economic growth in order to increase. Since education and GNI tend to be correlated, the five-variable model makes similar predictions as the two-variable model. The main difference appears to be in the speed with which democracy increases. Figure 7 shows how democracy is predicted to change in our four example countries. Here we see India reaching higher levels of democracy faster than in the two-variable model. Again, the variation in the prediction for democracy is high, and it is plausible that India reaches full democracy within 50 years.

**FIG. 8.**
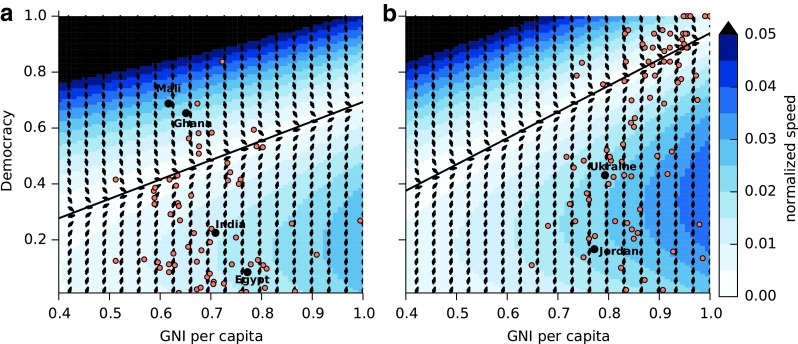
The phase portrait of the *G* and *D* subsystem with a scatter plot of the gross national income (GNI) per capita and democracy values for all countries in 2006 in the available dataset. The arrows show the direction of yearly change in the (*G,D*) vector and the color shows the magnitude of change assuming *Et*=0.473 the fixed point for *Et*. In **(a)** we show countries whose education indicator values *I* are below the global average of 0.621 and in **(b)** we show countries with *I* above 0.621. The phase portrait shows the estimated velocities in any year. The black lines show the characteristic curves for *D* given by (17) with points above the curve corresponding to negative change in *D* values and points below corresponding to positive changes, and these are computed by taking the average values of *I* for the countries in **(a)** and **(b)**, respectively.

Figure 8 shows a phase plane for democracy and GNI for two different cases: one in which education is relatively low *I*=0.42 (Fig. 8a) and the other for high education *I*=0.77 (Fig. 8b). The condition for increases in democracy, given by Equation (17), depends on *I*. Lower levels of education shift the condition for increasing democracy downward. By classifying the countries into those with education below the mean (Fig. 8a) and above the mean (Fig. 8b), we can see whether we predict an increase or decrease in democracy. India, Ukraine, Jordan, and Egypt all lie in an area where democracy is predicted to increase relatively rapidly in the near future. This observation is consistent with the simulations in Figure 7. These countries are on their way out of the trapped region and should move toward greater democracy in the future.

On the other hand, certain countries with exceptional levels of democracy are predicted to experience decreases. For instance, Mali and Ghana have exceptionally high levels of democracy given their levels of GNI. The suggestion here is that these countries would require extra support, in order to avoid the fate of those countries in the past (like Nigeria or Bangladesh, for instance), which experienced a decrease in democracy. Since the time the data was collected in 2006, Mali has followed the path suggested by the model, experiencing civil unrest and decreases in democracy.

Finally, we can calculate the rate at which points near the line given by Equation (17) move away from it, using the eigenvalue for these points. This eigenvalue is 0.002/*E*. Thus, a higher value of *E* actually results in slower movement through the “trap” region. This is because *E* slows down growth in *G*.

## Conclusions

In this article, we have extended the theoretical and empirical concept of the development trap studied in economics^3,17,18^ to the political dimension in terms of political regime development (democratization) and political culture development (emancipation). In line with more recent research,^30–33^ our analysis suggests that the development trap is a multidimensional phenomenon, because the economic and political spheres are closely linked and a development trap in the economic sphere necessarily translates into a development trap in the political sphere. Our contribution to this research field is the attempt to use the available data to quantify the development trap and factors causing it as well as factors contributing to overcome it. Moreover, we use the data to make probabilistic predictions about the time that would be necessary for a given country to overcome a certain development trap given its initial situation.

In this regard, we show how data-driven modeling and analysis can go beyond “black box” analysis and predictions.^3,6^ The analysis we do here reveals how different indicators interact and the implications of these interactions. This provides a step toward a method for elucidating theories about development directly from data.

It is widely recognized that the last 30 years have seen a global trend toward democratization, in terms of regime change and emancipation, where people become more tolerant of the rights of others.^12,13,34–36^ Despite this general trend, this article has shown that some countries may not experience democratization in the near future. They are caught in a development trap with respect to democracy and/or emancipation. Although in the long run (50–100 years) we expect to see democratic and emancipation changes in these countries too, in the near future these changes will happen only very slowly, with possible setbacks due to noisy fluctuations that are caused by various uncertainties.

Our analysis shows that countries are caught in a democracy trap if they have low levels of GNI and/or education. We quantified the democracy trap condition, Equation (17), and visualized the rate of change as a phase portrait (Fig. 8). Countries near the trap condition experience slow increases, or even decreases, in democracy. It is difficult to say for sure what will happen in a particular country, but the phase portrait, combined with the stochastic simulations, shows that these traps are a real possibility. For example, simulations for Egypt showed that full democracy is a possibility within the next 20 years, but so too is a long period of autocracy (low levels of democracy indicator).

We also found evidence for emancipation traps. Emancipation may be seen as the cultural representation of democratization.^12,34,37^ The data suggests that emancipation can only increase if life expectancy is above a certain threshold. As emancipation depends on both life expectancy and democracy, higher values of democracy contribute to a country's faster move through the trap region. This is an example of how being caught in one trap, that is, lack of democracy, can lead to a country being caught in another, that is, lack of emancipation.

Indicators can interact in other ways too. For example, an interesting result is that high levels of emancipation, prior to democratization, may actually slow down movement out of the trap region for democracy. This is because high emancipative values tend to slow down economic growth. However, since high levels of democracy are required for emancipation to grow, this may not prove possible in practice.

Given these dynamics and accounting for uncertainty in future events, we made probabilistic predictions about future democracy trajectories of four exemplary countries: Egypt, Jordan, India, and Ukraine. The five-variable model gives relatively “optimistic” predictions for all four countries in terms of democratization. The predictions generally suggest that all four countries are likely to become largely democratic within the next 50 years, but the trajectories are likely to be characterized by temporary setbacks. At certain points the changes may be extremely slow and give the impression that the country is stuck.

## Abbreviations Used

HDI: Human Development Index
GNI: gross national income
